# Paxillin expression is closely linked to the pathogenesis, progression and prognosis of gastric carcinomas

**DOI:** 10.3892/ol.2013.1686

**Published:** 2013-11-19

**Authors:** LI-JUN XIAO, EN-HONG ZHAO, SHUANG ZHAO, XIN ZHENG, HUA-CHUAN ZHENG, YASUO TAKANO, HONG-RU SONG

**Affiliations:** 1 Department of Immunology, Chengde Medical University, Chengde, Hebei 067000, P.R. China; 2 Third Surgical Department, Affiliated Hospital of Chengde Medical University, Chengde, Hebei 067000, P.R. China; 3 Clinical Cancer Institute, Kanagawa Cancer Center, Yokohama, Kanagawa 250-0134, Japan

**Keywords:** gastric carcinoma, tumorgenesis, paxillin, clinicopathological behaviors, prognosis

## Abstract

Paxillin encodes a focal adhesion-associated protein and is involved in the progression and aggressive phenotypes of malignancies through its interactions with the actin cytoskeleton and key signal transduction oncogenes. The present study aimed to investigate the clinicopathological and prognostic significance of paxillin in gastric cancer. The expression of paxillin was evaluated using tissue microarrays of gastric adjacent non-cancerous mucosa, adenoma and carcinoma specimens by immunohistochemistry. Paxillin expression was compared against clinicopathological parameters and the survival time of the patients. Paxillin was highly expressed in gastric adenoma compared with that in non-neoplastic mucosa and carcinoma (P<0.05). Paxillin expression was lower in the younger carcinoma patients compared with that in the elder carcinoma patients (P<0.05). Paxillin expression was negatively correlated with tumor size, depth of invasion and lymph node metastasis, but not with patient gender, lymphatic or venous invasion, or TNM staging (P>0.05). Higher paxillin expression was observed in intestinal-type compared with diffuse-type carcinoma (P<0.05). Kaplan-Meier analysis indicated a positive association between paxillin expression and cumulative survival rate in all, advanced and intestinal-type carcinoma patients (P<0.05). Multivariate analysis using the Cox proportional hazards model indicated that patient age, depth of invasion, lymphatic invasion, lymph node metastasis, TNM staging and Lauren classification were independent prognostic factors for all gastric carcinomas (P<0.05). Aberrant paxillin expression may be involved in the growth, invasion, metastasis and differentiation of gastric carcinoma. Altered paxillin expression may, therefore, be employed as an indicator of pathobiological behaviors and prognosis of gastric carcinomas.

## Introduction

Despite a global decline in the incidence and mortality of gastric cancer in the last 60 years, it remains the fourth most common and second most frequent cause of cancer-related mortality. Gastric cancer continues to be a major health concern due to the slow decrease in incidence in Asia and high mortality from diagnosed gastric carcinomas in the West, despite the advanced diagnostic and operative techniques that are commonly used in clinical practice (1,2). An increased understanding of the changes that occur in gene expression in gastric cancer, particularly the identification of novel biomarkers for cancer diagnosis and novel targets for treatment, is required for the improvement of diagnosis, treatment and prevention.

Paxillin is a focal adhesion-associated, phosphotyrosine-containing 68-kDa adaptor protein discovered in 1990 by Turner *et al*(3). Paxillin contains a number of motifs that mediate protein-protein interactions, including C-terminal LIM domains resembling a double zinc-finger domain, N-terminal LD motifs, SH3 and SH2 domain-binding sites, whose motifs serve as docking sites for cytoskeletal proteins, tyrosine kinases, serine/threonine kinases, GTPase activating proteins and other adaptor proteins that recruit additional enzymes into complex with paxillin (4). Multiple tyrosine, serine and threonine phosphorylation sites exist throughout the paxillin molecule, and are targeted by a diverse array of kinases that are activated in response to various adhesion stimuli and growth factors (PDGF, EGF and IL-3). These include p21-activated kinase, FAK-Src, receptor for activated C kinase 1, c-Jun N-terminal kinase, extracellular-signal-regulated kinase, p38 mitogen-activated protein kinase, cyclin-dependent kinase 5 and c-Abl. Paxillin is tyrosine-phosphorylated upon integrin engagement or growth factor stimulation, creating binding sites for the Crk adapter protein (5,6). Thus, paxillin may be involved in signal transduction, regulation of cell morphology and the recruitment of structural and signaling molecules to focal adhesions to control cell spread and migration (7,8).

Previous studies have demonstrated that paxillin was overexpressed in esophageal squamous cell carcinoma, lung carcinoma, breast cancer and prostate cancer (9–13). In breast cancer, it has been found that the overexpression of paxillin may represent a useful prognosticator and be employed to predict the clinical response to chemotherapy (12,14). To better understand the clinicopathological and prognostic significance of paxillin, we observed its expression in gastric non-neoplastic mucosa, adenoma and carcinoma using a combination of tissue microarray and immunohistochemistry. Paxillin expression was compared with the clinicopathological and prognostic features of gastric cancer.

## Materials and methods

### Patients

This retrospective study was carried out using curatively resected specimens of gastric cancer (n=392) and adjacent non-neoplastic mucosa (n=197) collected at Toyama University Hospital (Toyama, Japan) from 1993 to 2006. The adenoma samples were resected from endoscopic biopsy at Toyama University Hospital from 1997 to 2008. The patients with gastric carcinomas were 120 males and 272 females (38–88 years; mean, 66.7 years). Archival materials were obtained from the Department of Pathology of Toyama University Hospital. In 151 cases, tumor development was accompanied by lymph node metastasis. None of the patients underwent chemotherapy, radiotherapy and adjuvant treatment prior to surgery. All patients were followed up by consulting their case documents and by telephone.

### Pathology

All tissues were fixed in 10% neutralized formalin, embedded in paraffin, cut into 4-μm sections and stained with hematoxylin and eosin (H&E) in order to confirm the histological diagnosis and microscopic characteristics of the specimens. The staging for each gastric carcinoma was evaluated according to the Union for International Cancer Control system, which indicates the extent of tumor spread (15). Histological architecture was defined using the Lauren classification (16,17). The tumor size, depth of invasion, lymphatic and venous invasion, and lymph node metastasis of tumors were also determined.

### Tissue microarray (TMA)

From H&E-stained sections of the tumor cases, representative areas of solid tumor were selected for sampling and 2-mm diameter tissue cores per donor block were punched out and transferred to a recipient block with a maximum of 48 cores using a tissue microarrayer (KIN-1; Azumaya, Tokyo, Japan). Sections (4-μm) were consecutively cut from the microarrays and transferred to poly-lysine-coated glass slides.

### Immunohistochemistry

Serial sections of TMA were deparaffinized with xylene, rehydrated with alcohol, and subjected to immunohistochemical staining with intermittent microwave radiation, as previously described (18). Rabbit anti-human paxillin antibody (Epitomics, Inc., Burlingame, CA, USA) was used at 1:100 dilution to detect the respective proteins, with anti-rabbit Envison-PO (Dako, Carpinteria, CA, USA) as the secondary antibody. Binding was visualized with 3,3′-diaminobenzidine and counterstaining with Mayer’s hematoxylin was performed to aid orientation. Omission of the primary antibody was used as a negative control.

**Figure 1:**
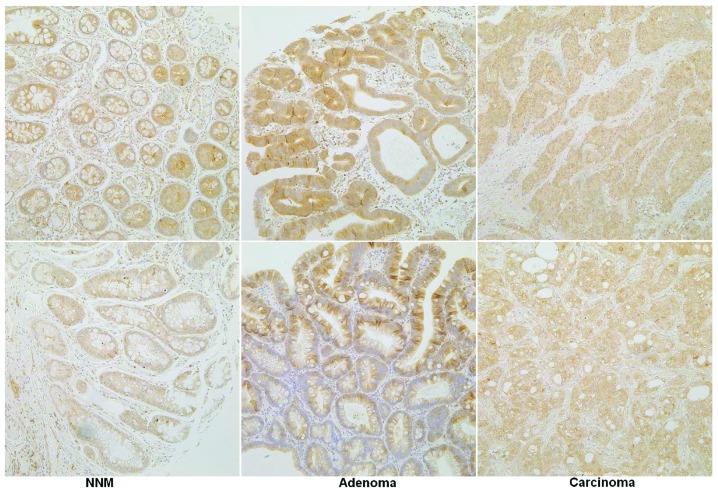
Immunohistochemical staining of gastric tissue samples showing the expression of paxillin. Strong positivity of paxillin was localized in the cytoplasm of gastric non-neoplasmic mucosa (NNM), adenoma and carcinoma (magnification, ×400).

Immunoreactivity for paxillin showed a cytoplasmic pattern (Fig. 1). One hundred cells were randomly selected and counted from five representative fields of each section, blindly, by three independent observers (L.J. Xiao and H.C. Zheng). The inconsistent data were confirmed by both observers until final agreements were reached. The expression positivity was graded and counted as follows: 0 = 0%; 1 = 1–49%; 2 = 50–74%; and 3≥ 75%. The staining intensity score was graded as follows: 1 = weak; 2 = intermediate; and 3 = strong. The scores for paxillin positivity and staining intensity were multiplied to obtain a final score, which determined their expression as − = 0; + = 1–2; ++ = 3–4; or +++ = 6–9.

### Statistical analysis

Statistical evaluation was performed using Spearman’s rank correlation test. Kaplan-Meier survival plots were generated and comparisons between survival curves were made with the log-rank test. Cox proportional hazards model was employed for multivariate analysis. SPSS 17.0 software (SPSS, Inc., Chicago, IL, USA) was applied to analyze all data, and P<0.05 was considered to indicate a statistically significant difference.

## Results

As indicated in Fig. 1, paxillin was positively expressed in the cytoplasm of gastric superficial epithelium, intestinal metaplasia, adenoma and carcinoma. The levels of paxillin expression was detected in gastric non-neoplastic mucosa (64.5%, 127/197), adenoma (92.3%, 60/67) and carcinoma (66.8%, 262/392), respectively. The expression of paxillin was significantly more highly expressed in gastric adenoma than in non-neoplastic mucosa and carcinoma (P<0.05, Table I). As shown in Table II, paxillin expression was negatively correlated with tumor size, depth of invasion, and lymph node metastasis, but not with gender, lymphatic or venous invasion, or TNM staging (P>0.05). Paxillin expression was higher in the elder carcinoma patients than in the younger carcinoma patients (P<0.05). There was higher paxillin expression in intestinal- compared with diffuse-type carcinoma (P<0.05).

**Figure 2:**
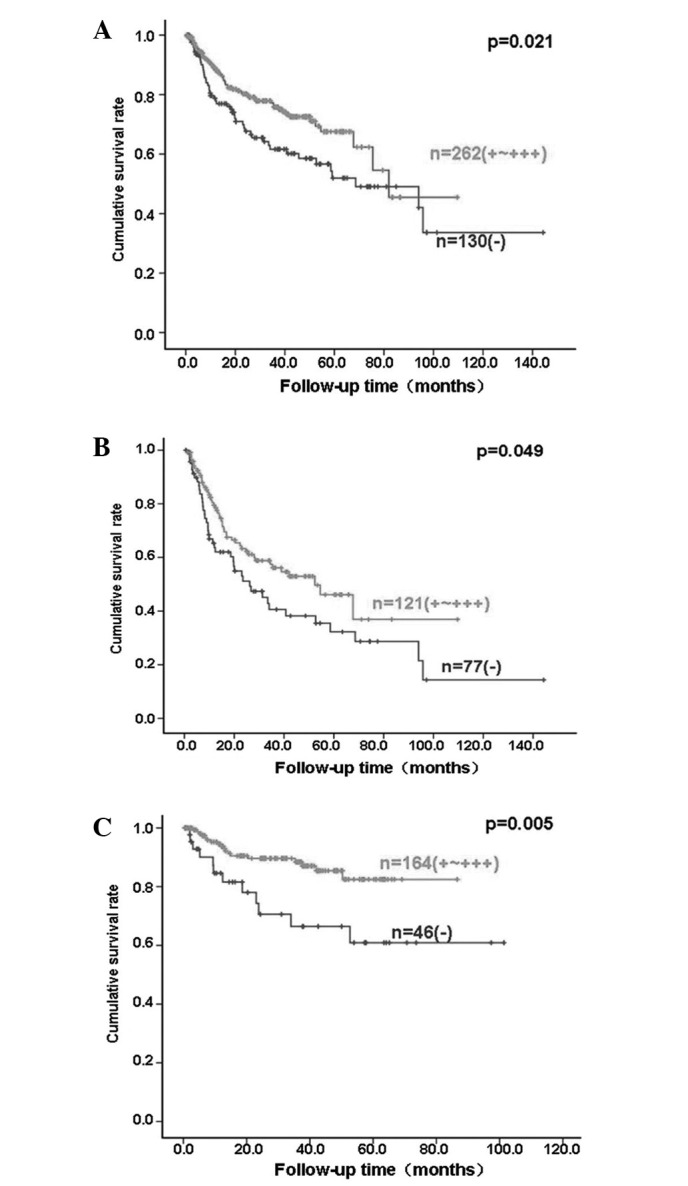
Prognostic significance of paxillin expression in patients with gastric cancer. Kaplan-Meier curves for cumulative survival rate of patients with (A) all, (B) advanced and (C) intestinal-type gastric carcinomas according to paxillin expression status.

Follow-up information was available on 392 of the gastric carcinoma patients for periods ranging from 0.2 months to 121 months (mean, 70.4 months). Fig. 2 shows survival curves stratified according to paxillin expression. Univariate analyses using the Kaplan-Meier method indicated a higher cumulative survival rate in all, advanced and intestinal-type carcinoma patients with weak, moderate and strong paxillin expression than in those without paxillin expression (P<0.05). Multivariate analysis using the Cox proportional hazards model indicated that patient age, depth of invasion, lymphatic invasion, lymph node metastasis, TNM staging and Lauren classification (P<0.05), but not patient gender, tumor size, venous invasion or paxillin expression (P>0.05), were independent prognostic factors for all gastric carcinomas (Table III).

## Discussion

Paxillin is a cytoskeletal protein that was recently identified as a component of focal adhesions and links between F-actin and integrin (19). In the present study, the cytoplasmic expression pattern of paxillin was observed in the gastric non-neoplastic epithelial cells, adenomas and adenocarcinomas. Statistically, paxillin expression was increased in gastric adenoma in comparison with that in the non-neoplastic mucosa and carcinoma. The adenoma can progress into and be incorporated with gastric well-differentiated carcinoma when it grows larger and *de novo* carcinogenesis is well understood, particularly in diffuse-type gastric carcinomas (20). These findings suggested that aberrant paxillin expression may be involved in the progression from gastric adenoma to adenocarcinoma. Higher paxillin expression in adenoma and intestinal-type carcinoma indicated that paxillin overexpression may be closely linked to the intestinal carcinogenic pathway of gastric cancer.

Cai *et al*(21) found that paxillin mRNA expression levels were significantly correlated with the differentiation degree, depth of invasion and lymph node metastasis of esophageal carcinoma. A previous study indicated that paxillin expression was correlated with distant metastasis and clinical stage of salivary adenoid cystic carcinoma (22). Li *et al*(23) documented that positive paxillin expression was significantly associated with low differentiation, with the presence of portal vein thrombosis, and with extra-hepatic metastasis of hepatocellular cell carcinoma. Li *et al*(24) found that paxillin positivity in human gastric cancer was associated with tumor stage, and siRNA targeting paxillin decreased the phosphorylation of paxillin (tyr118) and the invasiveness of AGS cells significantly as compared with controls. Previously, it was identified that overexpression of wild-type paxillin plasmids promoted cell proliferation and also enhanced migration, invasive capacity and metastasis of the colorectal cancer cells (25). However, paxillin expression was negatively correlated with tumor size, depth of invasion and lymph node metastasis of gastric cancer in the present study. The contradictory phenomena should be further investigated in the future.

Although all types of gastric cancer are malignant tumors that originate from the same gastric epithelium, the morphological features of the cancers vary substantially in individual patients. According to Lauren classification, gastric intestinal-type carcinoma is characterized by cohesive carcinoma cells that form gland-like tubular structures, such as well- and moderately differentiated carcinoma; while cell cohesion is less apparent or absent in diffuse-type carcinoma, such as poorly differentiated or signet ring cell carcinoma (16,17). Our findings demonstrated that paxillin was more highly expressed with a higher incidence in intestinal-type gastric cancer, which is presumed to arise from preceding dysplastic lesions, than diffuse-type ones, which evolve without any precedent dysplastic changes, indicating that distinct paxillin expression underlies the molecular mechanisms for the differentiation of intestinal- and diffuse-type carcinomas.

To date, there have been several studies describing the prognostic significance of paxillin expression in malignancies (11,12,14,26). In the present study, for the first time, we analyzed the correlation between paxillin expression and the survival rate of 392 patients with gastric carcinoma. The results revealed a close association between its overexpression and favorable survival. When stratified according to the depth of invasion, the significant correlation disappeared in the early gastric cancers, but not in the advanced ones, indicating that the association between paxillin expression and prognosis depends on the depth of invasion. The multivariate analysis demonstrated that patient age, depth of invasion, lymphatic invasion, lymph node metastasis, TNM staging and Lauren classification, but not patient gender, tumor size, venous invasion or paxillin expression, were independent prognostic factors for all gastric carcinomas. These findings suggested that paxillin expression is a good indicator for the favorable prognosis of gastric carcinoma patients, albeit it is not independent. By contrast, Li *et al*(11) found that no correlation occurred between expression of paxillin and patient survival of these patients with esophageal cancer. Zuo *et al*(26) found that paxillin expression was closely correlated with the prognosis of non-small cell lung carcinoma.

In conclusion, aberrant paxillin expression may be important in the malignant transformation of gastric epithelial cells. Its reduced expression was closely correlated with growth, invasion, metastasis and a worse prognosis of gastric carcinomas. Its expression may be employed to differentiate between the intestinal- and diffuse-type carcinomas. It was considered as a promising marker to indicate the pathobiological behaviors and prognosis of gastric carcinomas.

## Figures and Tables

**Table I:** Paxillin expression in gastric carcinomas.

		Paxillin expression				
			
Groups	n	−	+	++	+++	PR (%)
Non-cancerous mucosa	197	70	91	26	10	64.5
Adenoma	67	7	21	28	11	92.3^a^
Carcinoma	392	130	164	59	39	66.8

a P<0.001, compared with non-cancerous mucosa or carcinoma.

PR, positive rate.

**Table II:** Correlation between paxillin expression and clinicopathological features of gastric carcinomas.

		Paxillin expression					
				
Clinicopathological features	n	−	+	++	+++	PR (%)	P-value
Age (years)							0.027
<65	156	56	68	19	13	64.1	
≥65	236	74	96	40	26	68.6	
Gender							0.060
Male	272	89	105	46	32	67.3	
Female	120	41	59	13	7	65.8	
Tumor size (cm)							0.001
<4	204	58	80	40	26	71.6	
≥4	188	72	84	19	13	61.7	
Depth of invasion							<0.001
T_is_-_1_	200	59	85	35	21	70.5	
T_2–4_	192	71	79	24	18	63.0	
Lymphatic invasion							0.799
−	250	80	111	32	27	68.0	
+	142	50	53	27	12	64.8	
Venous invasion							
−	335	113	142	48	32	66.3	0.287
+	57	17	22	11	7	70.2	
Lymph node metastasis							
−	241	70	102	39	30	71.0	0.006
+	151	60	62	20	9	60.3	
UICC staging							0.352
0–I	215	68	89	36	22	68.4	
II–IV	177	62	75	23	17	65.0	
Lauren classification							<0.001
Intestinal type	210	46	98	36	30	78.1	
Diffuse type	172	80	62	22	8	53.5	

PR, positive rate; T_is_, carcinoma *in situ*; T_1_, lamina propria and submucosa; T_2_, muscularis propria and subserosa; T_3_, exposure to serosa; T_4_, invasion into serosa; UICC, Union for International Cancer Control.

**Table III:** Multivariate analysis of clinicopathological variables for survival with gastric carcinomas.

Clinicopathological parameters	Relative risk (95% CI)	P-value
Age (≥65 years)	1.902 (1.254–2.883)	0.002
Gender (male)	1.212 (0.750–1.959)	0.432
Tumor size (≥4 cm)	1.285 (0.771–2.141)	0.336
Depth of invasion (T_2–4_)	5.979 (2.084–17.152)	0.001
Lymphatic invasion (+)	1.995 (1.201–3.313)	0.008
Venous invasion (+)	1.202 (0.751–1.922)	0.444
Lymph node metastasis (+)	2.932 (1.535–5.602)	0.001
TNM staging (III–IV)	0.341 (0.119–0.974)	0.045
Lauren classification (diffuse type)	2.235 (1.396–3.577)	0.001
Paxillin expression (+~+++)	0.714 (0.475–1.073)	0.105

CI, confidence interval; T_2_, muscularis propria and subserosa; T_3_, exposure to serosa; T_4_, invasion into serosa; TNM, tumor-node-metastasis.
